# Blastocyst transfer for all? Higher cumulative live birth chance in a blastocyst-stage transfer policy compared to a cleavage-stage transfer policy

**Published:** 2019-06

**Authors:** I De Croo, R Colman, P De Sutter, K Tilleman

**Affiliations:** University Hospital Ghent Reproductive Medicine;; Biostatistics Unit, Ghent University Hospital, C. Heymanslaan 10, 9000 Ghent, Belgium.

**Keywords:** Cleavage-stage transfer, blastocyst-stage transfer, cumulative live birth rate

## Abstract

**Background:**

In an unselected patient population, what is the cumulative live birth rate per oocyte collection cycle in a blastocyst-stage transfer policy compared to a cleavage-stage transfer policy?

**Methods:**

A retrospective cohort analysis of 1656 IVF and ICSI cycles was performed in two timeframes between January 2010 and December 2016. Transfer was scheduled, either on day 3 (n=729) or on day 5 (n=927). In this study, the main outcome measure was cumulative live birth rate per oocyte collection cycle including fresh and frozen embryo transfers in both groups.

**Results:**

The cumulative live birth rates per oocyte collection cycle were comparable between patients with cleavage-stage transfers (day 3 group) and those with blastocyst-stage transfers (day 5 group) (23.7% versus 25.5%, respectively; p = 0.42). After controlling for confounders, there was a 34% increased chance of live birth with blastocyst-stage transfer policy compared with cleavage-stage transfer policy (odds ratio (OR) =1.34; 95% confidence interval (CI), 1.051 to 1.704; p = 0.018).

**Conclusion:**

In an unselected patient cohort, the cumulative live birth chance per oocyte collection cycle is higher in a blastocyst-stage transfer policy compared to a cleavage-stage transfer policy.

## Background

During the first two decades of In vitro fertilization (IVF), embryos were transferred at early cleavage stages. The introduction of blastocyst transfer in the mid 90’s significantly affected routine IVF. Transferring the embryo on day 5 or day 6 resulted in the exposure of the embryo to its natural environment since it is more analogous to the natural cycle. Additionally, extending the duration of the culture enables a better selection of embryos having a superior developmental capacity and thus a higher implantation potential (Marek et al., 1999). It has been shown that embryo transfer at the blastocyst stage increases the pregnancy rate per embryo transferred and this is especially relevant in the context of single embryo transfer (SET) policies intended to reduce multiple gestations (Papanikolaou et al., 2006). Although blastocyst transfer leads to higher live birth rates per embryo transfer (Glujovsky et al., 2016; Martins et al., 2017), there also have been safety concerns described: reports on a higher risk of preterm birth (Maheshwari et al., 2013; Dar et al., 2014), a study showed babies being large for gestational age (Mäkinen et al., 2013) and the prevalence of monozygotic twinning is reported to be higher in a blastocyst transfer policy compared to transfer at the cleavage stage (Sotiroska et al., 2015; Mateizel et al., 2016). In a recent meta-analysis, an increased incidence of transfer cancellations and a lower number of embryos cryopreserved have been shown to be associated with a blastocyst transfer policy (Martins et al., 2017). Based on these pessimistic outcomes, it is not surprising that blastocyst transfer has not yet become the strategy of choice for most clinics worldwide. However, recently, there is clearly a tendency to perform more blastocyst stage transfers. In Belgium, data from Belrap (Belgian Register for Reproductive Procreation) showed an increase from 30.2% blastocyst transfer in 2015 to 35.3% in 2016.

Embryo selection efforts have also made followers elsewhere: SET at blastocyst-stage has become a characteristic feature of Assisted Reproductive Technology (ART) in Australia/New Zealand (Chambers et al., 2015), areas of Canada (Bissonnette et al., 2011) and in Northern Europe (De Neubourg et al., 2013; Peeraer et al., 2014). Australia and New Zealand progressively shifted from cleavage- to blastocyst-stage embryo transfers, with blastocyst-stage transfers increasing from 49.8% in 2009 to 61.1% in 2013 (Kushnir et al., 2017).

Despite the lack of randomized control trials (RCT’s), blastocyst-stage transfer is seen to improve pregnancy and live birth rates as compared to cleavage-stage transfer (Glujovsky et al., 2016). Unfortunately, no study to date has answered superiority of cumulative live birth rates per oocyte collection cycle for a blastocyst transfer policy. Therefore, many centers are reluctant to decide between a cleavage-stage or blastocyst-stage policy and often proceed to the transfer of a blastocyst only in good prognosis patients. Previous studies on cumulative live birth rates in day 5 transfer regimes are solely established on a selected patient cohort based on age (Rienzi et al., 2002; De Vos et al., 2016) or on available zygotes (Rienzi et al., 2002; Fernández-Shaw et al., 2015). It therefore remains questionable whether a blastocyst-stage approach is indeed beneficial for all patients seeking ART.

The aim of this retrospective analysis was to analyze the cumulative live birth rates after cleavage-stage and blastocyst-stage transfer in an unselected population, combining both fresh and frozen embryo transfers.

## Materials and methods

### Study design

A retrospective single center study was performed in the Ghent University Hospital. IVF and Intracytoplasmic Sperm Injection (ICSI)-cycles were analyzed in two timeframes, a first period was situated between January 2010 and December 2011 during which all embryo transfers were performed on day 3 and supernumerary embryos were frozen with a slow freezing protocol on day 3 (day 3 group). A second period was situated between January 2016 and December 2016 during which all embryo transfers were performed on day 5 and supernumerary embryos vitrified on day 5 (day 5 group).

The day of transfer was a time depending decision. Before January 2012, all embryo transfers were performed at the cleavage-stage and supernumerary embryos were frozen with a slow freezing protocol. From January 2012 on, a stepwise approach was chosen to switch from a cleavage-stage transfer policy to a blastocyst-stage transfer policy. All supernumerary embryos were left in culture until day 5 and were vitrified as blastocysts. Patients treated between January 2012 to December 2013 and having more than 9 zygotes on day 1 had a transfer on day 5, patients with less than 10 zygotes had a transfer on day 3. Patients treated between January 2014 and December 2015 and having more than 4 zygotes on day 1 had a transfer on day 5, patients with less than 5 zygotes had a transfer on day 3. From January 2016, all patients had a transfer on day 5.

Oocyte collection cycles performed between January 2012 and December 2015 were thus excluded because of the mixed transfer policy. Day 2 transfers were also excluded from the analysis. The sperm used for IVF or ICSI were either fresh or frozen partner ejaculates, surgically retrieved spermatozoa, or frozen donor ejaculates. Cycles with Pre-implantation Genetic testing (PGT), oocyte donation and elective freeze-all cycles were excluded from the analysis.

If the fresh transfer was unsuccessful, a thawing/warming transfer cycle was performed before starting a new oocyte collection cycle.

In the day 3 group, the outcome of the consecutive frozen-warming cycles was analyzed until December 2012 (fresh cycles between January 2010 and December 2011)

In the day 5 group, the outcome of the consecutive vitrification-warming cycles was analyzed until December 2017 (fresh cycles between January 2016 and December 2016).

The main outcome measure of this analysis was cumulative live birth rate per oocyte collection cycle including fresh and frozen embryo transfers in both groups until the birth of a first child.

### Ovarian stimulation and oocyte retrieval

For pituitary down-regulation three protocols (two agonist, one antagonist) were used. The short agonist protocol started following at least 14 days ethinylestradiol 50/ levonorgestrel 150 (M50) (Microgynon ’50’ ® ; Bayer Pharma AG, Berlin, Germany). After ending of M50 (“day 0” of the IVF-cycle) a Gonadotropin-Releasing Hormone agonist (GnRH-a) (Triptorelin; Decapeptyl ® ; Ferring, Hoofddorp, The Netherlands) was started on day 3 until LH or HCG administration. Gonadotropins (FSH: Gonal-F ® , Serono Benelux, London, UK; or Puregon R; MSD, Oss, the Netherlands or human menopausal gonadotropin: Menopur ® ; Ferring, Hoofddorp, The Netherlands) were added starting on day 5. The longer agonist protocol started using Decapeptyl depot on day 21 of the previous natural menstrual cycle. After at least 14 days GnRH-a pre-treatment additional gonadotropin administration was started. In both agonist protocols, controlled ovarian hyper-stimulation was achieved using daily doses between 150 IU and 300 IU of gonadotropins. For the antagonist protocol, gonadotropins were started on day 3 of the natural menstrual cycle and a GnRH-antagonist (Cetrorelix 0.25mg; Cetrotide ® , Merck Serono, Geneva, Switzerland) was injected subcutaneously as a daily dose from cycle day 6 until the day prior to oocyte retrieval.

Between 34 to 36 hours after the human chorionic gonadotropin (hCG) (Pregnyl 5.000IU ® ; MSD Oss, the Netherlands) or recombinant hCG (Ovitrelle ® 6500IU, Serono Benelux, London, UK) injection, follicle aspiration was performed. To support the luteal phase, all women were treated with intravaginal progesterone (Utrogestan ® , Besins Healthcare, Brussels, Belgium) starting on the day of human chorionic gonadotropin (hCG) or recombinant LH injection. A serum-hCG test was performed sixteen days after oocyte retrieval.

### Fertilization, embryo culture and fresh embryo transfer

Conventional IVF and ICSI procedures were carried out 3-6 hours after oocyte retrieval. Oocytes and embryos were cultured individually in sequential media (Cleavage and Blastocyst medium, Cook, USA) in microdroplets under oil (Irvine Scientific, Ireland) in a 6% CO 2 , 5% O 2 and 89% N 2 incubator at 37°C (Binder 210, VWR, Belgium).

Fertilization was assessed 16-19h after insemination or ICSI. The morphological evaluation of embryo development was performed daily until the day of transfer.

Briefly, the embryo quality on day 2 and day 3 was assessed on the basis of the number of blastomeres, the rate of fragmentation and the presence of multi-nucleation. On day four, the evaluation included assessment of the compaction stage.

Assessment on day 5 was based on the classification system of Gardner and Schoolcraft (1999), where the embryo is ideally developed to the blastocyst stage. First the expansion status and hatching were scored (1 = early blastocyst, blastocoele is less than half of the embryo volume, 2 = blastocyst, blastocoele is half or more than half of the embryo volume, 3 = blastocoele completely fills the embryo, 4 = expanded blastocyst and 5 = hatching blastocyst). If the score of the blastocyst stage was >2; the inner cell mass (ICM) was characterized (A = tightly packed, many cells, B = loosely grouped, several cells and C = very few cells) and trophectoderm (TE) was evaluated (A = many cells forming a cohesive epithelium, B = few cells forming a loose epithelium and C = very few, large cells).

### Slow freezing, vitrification and frozen embryo transfer

In the day 3 group, supernumerary day 3 embryos with at least six blastomeres and <30% fragmentation were cryopreserved using slow freezing protocol. Briefly, the slow freezing protocol was performed using CBS High-Security straws (CryoBiosSystem, L’aigle, France) with 1,2-propanediol-sucrose as cryoprotectant (Sydney IVF cryopreservation kit, Cook, USA).

In the day 5 group, supernumerary blastocysts with at least expansion stage 1, inner cell mass score A, B or C and trophectoderm score A, B or C were cryopreserved on day 5. The vitrification procedure was performed using CBS-VIT High-Security straws (CryoBiosSystem, L’aigle, France) with dimethylsulphoxide-ethylene glycol-sucrose as the cryoprotectant (Irvine Scientific Vit Kit-Freeze, Ireland).

The patients having regular ovulatory cycles underwent a frozen embryo transfer (FET ) in a natural cycle (natural cycles). During natural cycles patients were monitored with transvaginal ultrasound and serum estradiol (E2) and luteinizing hormone (LH) concentrations. In case patients did not have a regular ovulatory cycle, endometrial preparation was initiated by oral administration of 6 to 12 mg estradiol valerate dd (Progynova ® , Bayer, Belgium) until and when the endometrial thickness was >6mm on transvaginal ultrasound (artificial cycles). At that moment 3x200 mg dd micronized progesterone vaginally (Utrogestan ® , Besins, Belgium) was added to the daily oral estradiol intake. The first day of start of progesterone application was set as day zero for calculating the day of thawing.

The transfer was performed on the 4th or 6th day after ovulation depending on the day of cryopreservation (day 3 or day 5, respectively). Embryos were thawed one day before transfer and a maximum of two embryos were replaced. All transfers were performed using a Cook embryo replacement catheter (Sydney IVF, Cook, USA).

### Outcomes and statistical analysis

Cumulative live birth was the primary outcome measure of this study (Zegers-Hochschild et al., 2017). Relevant data for all cycles were extracted from electronic patient records and recorded in a database. Normally distributed data were expressed as mean ± standard deviation (SD). Wilcoxon tests for independents samples were applied to compare the number of embryos transferred between day 3 and day 5. Cumulative incidence plots were created for the event “Live birth”. Fisher’s exact tests were used to compare success rates (“Live birth”) between day 3 and day 5. Parameters identified as significant were entered in a multiple logistic regression in order to adjust the predicted cumulative live birth rates. Two tailed p<0.05 was considered statistically significant. Statistical analysis was performed using R version 3.5.1.

## Results

### Trial population

**Figure 1:**
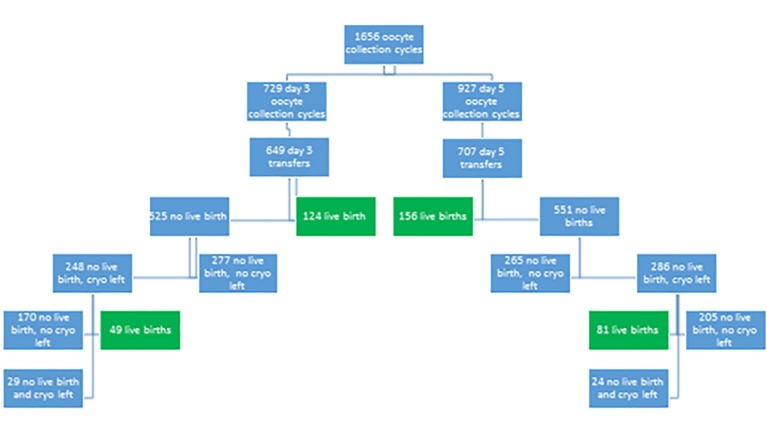
Flow chart of the study

The present study included a total of 1656 fresh IVF and ICSI oocyte collection cycles (Table I and Figure 1). A cleavage-stage transfer (day 3 group) was scheduled in 729 cycles and a blastocyst-stage transfer (day 5 group) was scheduled in 927 cycles. Women in the day 5 group were significantly older than in the day 3 group (35.5 ± 4.9 versus 34.5 ± 5.0, respectively; p = 0.030). The partner’s age was also significantly higher in the day 5 group as compared to the day 3 group (39.2 ± 8.1 versus 38.1 ± 7.5, respectively; p = 0.011).

**Table I:** — Patient and treatment cycle characteristics in a comparison of cumulative live birth between cleavage-stage and blastocyst-stage embryo transfer.

		Day 3	Day 5	P value
Fresh cycles		729	927	
Female age (years)		34.5 ± 5.0	35.0 ± 4.9	0.030
Male age (years)		38.1 ± 7.5	39.2 ± 8.1	0.011
Stimulation protocol				0.003
	Agonist	572 (78.5%)	745 (80.4%)	
	Antagonist	157 (21.5%)	182 (19.6%)	
Total dose gonadotrophins		2582 ± 1427	2861 ± 1145	<0.001
Oocytes retrieved		10.9 ± 7.3	9.6 ± 5.10	<0.001
Oocytes fertilized		6.0 ± 4.8	4.9 ± 3.5	<0.001
Embryos cryopreserved		3.7 ± 3.0	2.7 ± 1.8	<0.001
Transfer rate (%)		649/729 (89%)	707/927 (76.3%)	<0.001
No transfer because of bad embryo quality		21/729 (2.9%)	174/927 (18.8%)	<0.001
Embryos transferred fresh		1.63 ± 0.71	1.16 ± 0.38	<0.001
Fresh SET		314/649 (48.4%)	597/707 (84.4%)	<0.001
Frozen transfer rate (%)		331/789 (41.9%)	474/636 (74.5%)	<0.001
Frozen SET		153/233 (65.7%)	431/458 (94.1%)	<0.001

SET : single embryo transfer. Continuous data are presented as mean ± SD and compared using the independent Student’s t-test. Categorical data are expressed as their frequency and percentage within each study group and compared using the Fisher’s exact test.

The total dose of gonadotrophins was significantly higher in the day 5 group as compared to the day 3 group (2861 ± 1145 versus 2582 ± 1427, respectively; p < 0.001). Comparable proportions of patients underwent an agonist or antagonist stimulation protocol.

Patients in the day 3 group had significantly more oocytes retrieved than women in the day 5 group (10.9 ± 7.3 versus 9.6 ± 5.1, respectively; p < 0.001) and more oocytes were fertilized (6.0 ± 4.8 versus 4.9 ± 3.5, respectively; p < 0.001). The number of embryos cryopreserved per patient was significantly higher in the day 3 group compared to the number of blastocysts vitrified in the day 5 group (3.7 ± 3.0 versus 2.7 ± 1.8, respectively; p < 0.001).

In the day 3 group, 89.0% of the patients had a fresh transfer which was significantly higher compared to the day 5 group (76.3%; p<0.001). In the day 5 group, 174 patients did not have any embryos that reached the blastocyst stage to perform the embryo transfer; in the day 3 group only 21 patients did not have an embryo transfer because of bad embryo quality (18.8% versus 2.9%, respectively; p < 0.001).

A significantly higher number of fresh single embryo transfers (SET) were performed in the day 5 group as compared to the day 3 group (84.4% versus 48.4%, respectively; p < 0.001). The mean number of embryos transferred in a fresh transfer was significantly higher for day 3 as compared to day 5 (1.63 ± 0.71 versus 1.16 ± 0.38, respectively; p < 0.001). Transfer rates were significantly higher after warming of blastocysts-stage embryos than after thawing of cleavage-stage embryos (74.5% versus 41.9%, respectively; p <0.001). The percentage SET in frozen cycles was also significantly lower in the day 3 group as compared to the day 5 group (63.7% versus 95.8%, respectively; p<0.001).

### Live birth outcome

Despite the lower fresh transfer rate in the day 5 group, live birth rate per oocyte collection cycle did not differ between day 5 and day 3 (16.8% versus 17.0%, respectively; p = 0.947) (Table II). The miscarriage rate after transfer on day 3 was 23.1% which was similar after transfer on day 5 (28.3%). The twin rate per live birth was higher in the day 3 group than in the day 5 group (9.7% versus 4.5%; p=0.098) but did not reach significance.

**Table II:** — Treatment cycle live birth outcome.

		Day 3	Day 5	P value
Fresh cycles		729	927	
Fresh transfers		649	707	0.030
Live birth per fresh transfer		124/649 (19.1%)	156/707 (22.1%)	0.180
Live births per oocyte collection		124/729 (17.0%)	156/927 (16.8%)	0.947
	Singletons	112	149	
	Twins	12 (9.7%)	7 (4.5%)	0.098
Miscarriage rate after fresh transfer		40/173 (23.1%)	77/272 (28.3%)	0.269
Patients with no live birth after fresh transfer and cryopreserved embryos left		248/525 (47.2%)	286/551 (51.9%)	0.128
Live birth from frozen transfers		49/233(21.0%)	81/458 (17.6%)	0.304
	Singletons	37	78	
	Twins	12 (24.5%)	3 (3.7%)	<0.001
Cumulative live birth per oocyte collection cycle		173/729 (23.7%)	237/927 (25.5%)	0.422
Cumulative live birth per fresh transfer		173/649 (26.7%)	237/707 (33.5%)	0.006
Cycles with no live birth and no cryopreserved embryos left		527/729 (72.3%)	666/927 (71.8%)	0.869

Categorical data are expressed as their frequency and percentage within each study group and compared using the Fisher’s exact test. Miscarriage rate : Number of miscarriages per number of pos hCG after fresh transfer

The percentage of patients not achieving a live birth after fresh embryo transfer and still having cryopreserved embryos left, for subsequent thawing/warming cycles, was similar in both groups (47.2% for day 3 versus 51.9% for day 5; p = 0.128) (Figure 1).

Live birth rate from frozen transfers of patients having an unsuccessful fresh embryo transfer was similar for both groups (21.0% for day 3 versus 17.6% for day 5). However, multiple pregnancy rate in frozen transfers was significantly higher in the day 3 group as compared to day 5 group (24.5% versus 3.7%, respectively; p < 0.001).

The percentage of patients without a live birth and without cryopreserved embryos left was similar in both groups (72.3% versus 71.8%; p=0.869) (Table I).

At the end of the study cascade, the number of patients without live birth and still have embryos cryopreserved was 29 in the day 3 group and 24 in the day 5 group (Figure 1).

### Cumulative live birth rates per oocyte collection cycle

**Figure 2:**
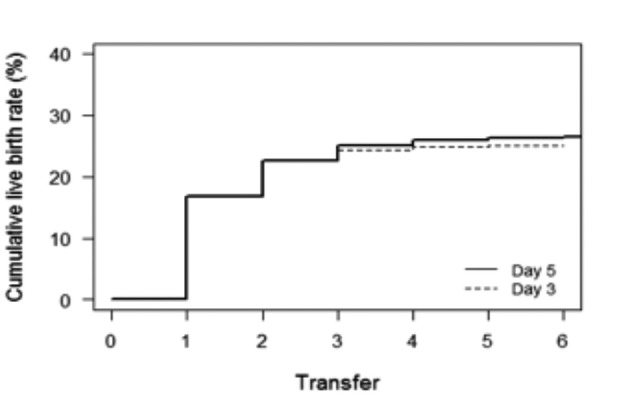
Cumulative incidence plot: Cumulative live birth rates per oocyte collection cycle in relation to transfer day. Graph depicts the time to pregnancy, expressed by the number of embryo transfers required.

The cumulative live birth rates per oocyte collection cycle were comparable between cleavage-stage transfers (day 3 group) and blastocyst-stage transfers (day 5 group) (23.7% versus 25.5%, respectively; p=0.42) (Table II). After adjustment for the differences in female age and total number of oocytes retrieved, the estimated odds of having a live birth per oocyte collection cycle were 33% higher for day 5 as for day 3 (Figure 2, Table III) (OR =1.338 , 95% CI=1.051 to 1.704, p=0.018).

**Table III:** — Multiple logistic regression for cumulative live birth rates per oocyte collection cycle.

		Adjusted OR (95% CI)	P value
Embryo stage			
	Day 3	Reference	
	Day 5	1.338 (1.051 to 1.704)	0.018
Female age		0.911 (0.889 to 0.935)	<0.001
Oocytes retrieved		1.077 (1.057 to 1.098)	<0.001

OR, odds ratio; CI, confidence interval; Multivariate mixed-effects logistic regression for cumulative live birth accounting for the day of transfer, female age and number of oocytes retrieved.

### Cumulative live birth rates per fresh transfer

**Figure 3:**
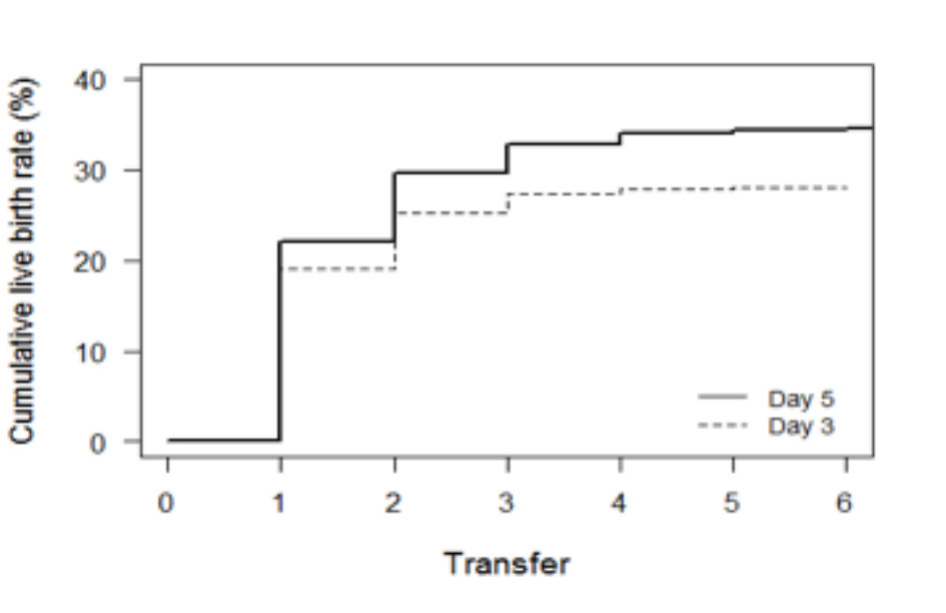
Cumulative live birth rates per fresh transfer. Graph depicts the time to pregnancy,expressed by the number of embryo transfers required

The cumulative live birth rate was significantly higher for day 5 as compared to day 3 (33.6% versus 26.7%; p=0.006) once patients received a fresh transfer. The results for live birth rates remained significant after controlling for female age, total number of oocytes retrieved and number of embryos transferred (OR=1.520, 95% CI= 1.129 to 2.048, P=0.006). The estimated odds of having a live birth are 52% higher for day 5 compared to day 3 if patients had a fresh transfer (Figure 3, Table IV) (OR =1.520, 95% CI=1.129 to 2.048 , p=0.006).

**Table IV:** — Multiple logistic regression for cumulative live birth rates per fresh transfer.

		Adjusted OR (95% CI)	P value
Embryo stage			
	Day 3	Reference	
	Day 5	1.520 (1.129 to 2.048)	0.006
Female age		0.929 (0.902 to 0.956)	<0.001
Oocytes retrieved		1.021 (0.999 to 1.043)	0.06
Number of embryos transferred			
	One	Reference	
	Two or more	1.649 (1.192 to 2.281)	0.003

## Discussion

The present retrospective analysis showed a higher cumulative live birth chance per oocyte collection cycle in a blastocyst-stage transfer policy compared to a cleavage-stage transfer policy and this in an unselected patient population.

We considered this study necessary, as there is currently insufficient evidence to decide whether to opt for a cleavage-stage or blastocyst-stage transfer policy in IVF. There is an ongoing debate about the benefit of blastocyst transfer and RCT’s, including the outcome of subsequent frozen cycles. Our study showed no evidence of differences in cumulative live birth rates after cleavage-stage and blastocyst-stage transfer from one single oocyte retrieval cycle. We reported a cumulative live birth rate of 23.7% after cleavage-stage transfer and 25.5% after blastocyst-stage transfer per oocyte collection cycle. This is a confirmation of earlier publications where no difference in cumulative live birth rate per cycle was found between blastocyst-stage and cleavage-stage transfer (Fernández-Shaw et al., 2015, De Vos et al., 2016). However, when controlling for confounders, a blastocyst-stage transfer policy was associated with a 34% increased odds of live birth per oocyte collection cycle. During this timeframe, the patients who did not achieve a live birth and still have cryopreserved embryos (29/729) or blastocysts (24/927) in stock are believed not to influence the final chance of having a live birth.

The inclusion of this large cohort of unselected patients forms both the strength and the weakness of this study. An important limitation of this study, is the length of the study itself. Indeed, during the studied period we covered data from two periods with a gap of five years because we performed a stepwise approach to change from a cleavage-stage transfer policy to blastocyst-stage transfer policy as described in the study design. Despite the long period (2010-2018) of the analysis, the laboratory conditions in terms of sequential media, oil, incubators remained the same and the laboratory was situated in a cleanroom environment with steady environmental monitoring. Previous studies all made a selection of patients based on age (Rienzi et al., 2002; De Vos et al., 2016) or on available zygotes (Rienzi et al., 2002; Fernández-Shaw et al., 2015) which does not reflect the daily clinical practice.

It was already established that blastocyst-stage transfer was associated with higher implantation rates per transferred embryo, and thus with higher pregnancy rates per fresh transfer than cleavage-stage transfer (Glujovsky et al., 2016). Also in our study, the estimated odds of having a live birth in patients who had a fresh transfer on day 5 was 52% higher (p=0.006) than patients who had a fresh transfer on day 3. So, once patients had an embryo that could be transferred in a fresh transfer cycle, the cumulative live birth rate was significantly higher for day 5 (26.7%) as for day 3 (33.5%) even after adjusting for differences in female age, number of oocytes retrieved and number of embryos transferred. This is in agreement with the conclusion of the systematic review of Glujovsky et al. (2016) where it was stated that although there was a benefit favoring blastocyst transfer in fresh cycles, it remained unclear whether the day of transfer would impact on the cumulative rate.

The aim of extending embryo culture to the blastocyst stage allows for a better embryo selection. This is confirmed in our analysis by a significantly lower transfer rate and smaller number of embryos cryopreserved in day 5 transfer group and is in agreement with earlier publications (Van Der Auwera et al., 2002; Guerif et al., 2009). Avoiding unnecessary embryo transfers needs to be balanced against the need for an additional oocyte retrieval. Some advocate that it is better for women to hear that their embryos failed to develop to blastocyst than go through with transfer of a cleavage stage embryo having a low potential to implant. However, very little is known of the emotional status of couples or women presented with such choices (Zemyarska, 2019).

In conclusion, this retrospective study conducted in an unselected patient cohort, showed similar cumulative live birth rates per oocyte collection cycle between a day 3 or day 5 transfer policy, even though fewer embryos were required to achieve the first live birth delivery in a blastocyst transfer policy. In the subpopulation of patients having a fresh transfer, the cumulative live birth rate was significantly higher for day 5 compared to day 3. Because of the higher transfer cancellation rate in the fresh cycle, a blastocyst transfer policy allows to be more cost-efficient as more unnecessary transfers can be avoided. This could also contribute to a reduction of the emotional stress of infertile couples and cost saving to both the patients and the health care system. When starting an IVF cycle in a day 5 transfer policy, patients will have a higher cumulative live birth chance than in a day 3 transfer policy. In the case that the cycle is unsuccessful because there are no embryos that have reached the blastocyst stage, patients will have the benefit of starting a subsequent cycle sooner rather than later. Although, a difficult message to communicate for the medical staff and a huge disappointment for the patients themselves, knowing this sooner than later, augments the efficiency and the quality of ART.

Even after this study and presenting these results, many centers might not change their transfer policy to a day 5 transfer for all. Still, the debate continues and maybe for some poor prognosis patient groups a day 3 transfer would still be beneficiary. Only an RCT can truly give the answer to this question and this study is still lacking in literature. Although the study design of such an RCT is quite straight forward, many other factors play a role in executing the study: the emotional burden for patients where a randomization is deciding the transfer policy instead of a shared decision and the extensive counseling that goes along with it. Additionally, the reimbursement policy of IVF treatments has its impact. In Belgium, patients get up to 6 IVF cycles reimbursed, thus, it is imaginable that in countries where there is limited or no reimbursement transfer policies might be influenced by the financial policy too.

Changing an embryo transfer policy is not easy and many factors have an impact on the policy chosen by each center. We believe, that this study contributes significantly to the lively debate in ART on whether to transfer on day 3 or day 5.
